# Heterogeneity of breast cancer risk within the South Asian female population in England: a population-based case–control study of first-generation migrants

**DOI:** 10.1038/sj.bjc.6601440

**Published:** 2004-01-06

**Authors:** V A McCormack, P Mangtani, D Bhakta, A J McMichael, I dos Santos Silva

**Affiliations:** 1 Department of Epidemiology and Population Health, London School of Hygiene and Tropical Medicine, London WC1E 7HT, UK

**Keywords:** breast cancer, South Asian, ethnicity

## Abstract

South Asian women in England have a lower breast cancer risk than their English-native counterparts, but less is known about variations in risk between distinct South Asian ethnic subgroups. We used the data from a population-based case–control study of first-generation South Asian migrants to assess risks by ethnic subgroup. In all, 240 breast cancer cases, identified through cancer registries, were individually matched on age and general practitioner to two controls. Information on the region of origin, religious and linguistic background, and on breast cancer risk factors was obtained from participants. Breast cancer odds varied significantly between the ethnic subgroups (*P*=0.008), with risk increasing in the following order: Bangladeshi Muslims (odds ratio (OR) 0.33, 95% confidence interval (CI): 0.10, 1.06), Punjabi Hindu (OR 0.59, 95% CI: 0.33, 1.27), Gujarati Hindu (1=reference group), Punjabi Sikh (OR 1.23, 95% CI: 0.72, 2.11) and Pakistani/Indian Muslims (OR 1.76, 95% CI: 1.10, 2.81). The statistically significant raised risk in Pakistani/Indian Muslims increased with adjustment for socioeconomic and reproductive risk factors (OR 2.12, 95% CI: 1.25, 3.58), but was attenuated, and no longer significant, with further adjustment for waist circumference and intake of nonstarch polysaccharides and fat (OR 1.49, 95% CI: 0.85, 2.63). These findings reveal differences in breast cancer risk between South Asian ethnic subgroups, which were not fully explained by reproductive differences, but were partly accounted for by diet and body size.

Breast cancer incidence rates in South Asian women living in England are lower than those in the English native population, but higher than those in the Indian subcontinent (Harding and Rosato, 1999; Winter *et al*, 1999; Smith *et al*, 2003), consistent with studies in migrants from low- to high-risk countries in which incidence approaches that of the native population within one or two generations (Shimizu *et al*, 1991; Ziegler *et al*, 1993). Although rates are lower than that of the native population, breast cancer remains the most common cancer among South Asian women (Harding and Rosato, 1999; Winter *et al*, 1999).

Breast cancer rates in this ethnic group, however, reflect the average experience of this diverse population, and thus may conceal important within-population differences in risk. The South Asian population comprises several ethnic subgroups that can vary greatly in terms of culture, religious practices and lifestyle. These variations may lead to differences in the prevalence of breast cancer risk factors and thus in breast cancer risk. Many Gujarati Hindu women, for example, are vegetarians, whereas the majority of Pakistani Muslim women are meat-eaters and the latter, on average, have higher parity. There is also evidence from the Indian subcontinent of variations in risk by religion (Jussawalla *et al*, 1985) and region (Yeole *et al*, 1990; Bhurgri *et al*, 2000; Sen *et al*, 2002), but, to our knowledge, only one study of mortality has explored whether such differences persist among South Asians in the UK (Balarajan *et al*, 1984). However, the lack of adequate information on ethnicity in cancer registration data has hampered the examination of incidence rates by ethnic group (Harding and Allen, 1996).

We examined variations in breast cancer risk by ethnic subgroup in a population-based case–control study conducted among first-generation South Asian migrant women in England, and investigated to what extent these variations may be explained by established breast cancer risk factors.

## MATERIAL AND METHODS

The present study is based on data from a case–control study that was designed with the primary aim of investigating the relationship between dietary intake and breast cancer among South Asian migrant women in England (dos Santos Silva *et al*, 2002). In brief, potential cases were identified from the Thames and West Midlands population-based cancer registries during 1995–99 as women of South Asian ethnic origin with breast cancer diagnosed in the previous 2 years, under age 75 years, and were living at the time of the study. For each case, two controls without cancer, matched on date of birth within 5 years, were randomly selected from the South Asian women who were registered with the index case's general practitioner (GP). Women of South Asian ethnicity were identified using a validated name analysis technique (Nanchahal *et al*, 2001) and were included if they were women born in East Africa or the Indian subcontinent. South Asian ethnicity was later confirmed with their GPs and the participants themselves, who also provided information on the country/region of origin and religious background.

After obtaining permission from the relevant GP, participants were invited to be interviewed at home in English or in their mother tongue, using a structured questionnaire. The women were asked about reproductive factors, including age at menarche, pregnancies, breast-feeding practices and menopausal status. They were considered to be postmenopausal if they had stopped menstruating for at least 6 months. The women were asked about their family history of breast and other cancers, and whether their parents were first-degree relatives. The interviewer also administered a food frequency questionnaire (FFQ), obtaining information about the quantity/portion size and frequency of consumption of 207 food items 2–3 years previously. The average daily nutrient intake was calculated in CompEat (Nutrition Systems, 1995) using a nutritional database, which included McCance and Widdowson 5th edition (Holland *et al*, 1995) and its supplements, as well as foods commonly consumed by South Asians in the UK (Judd *et al*, 2000). The FFQ was validated in a subset of controls using 12-monthly 24-h dietary recalls as the reference method, and the percentages of women classified in the same ±1 quartile of intake distribution by both methods were 74% for total energy and 85, 96 and 90% for energy-adjusted fat, carbohydrates, protein and NSP, respectively (Sevak *et al*, in press). At the end of the interview, the women were weighed (to the nearest 0.1 kg), and the standing height, sitting height and hip and waist circumferences were measured to the nearest 0.1 cm, using standardised procedures (dos Santos Silva *et al*, 2002). Levels of physical activity at ages 12 and 30 years were assessed using a series of questions on the duration and frequency of leisure, occupation-related and housework activities. These questions were used to calculate the metabolic equivalent (MET) score per week for each individual (Ainsworth *et al*, 1993); this corresponds to the energy expenditure per week expressed as multiples of the resting metabolic rate (no. hours × intensity rate/resting metabolic rate).

We categorised the participants into six relatively homogeneous subgroups according to religion, mother tongue and birth place (both country and province) as follows (*n*=number of cases and controls):

1. *Gujarati Hindu* (*n*=263): Hindu women born in Gujarat, India (38%), or born in East Africa, but whose parents came from Gujarat, India (27%), and Hindu women from other provinces of India (except Punjab) whose mother tongue was Hindi/Gujarati (35%).
2. *Punjabi Hindu* (*n*=72): Hindu women born in Punjab, India (58%), or born in East Africa, but whose parents came from Punjab (13%) and Hindu women from other Indian provinces whose mother tongue was Punjabi (29%).
3. *Punjabi Sikh* (*n*=163): Sikh women whose mother tongue was Punjabi, regardless of the place of birth. In all, 74% were born in Punjab, 15% in other provinces of India and 11% in East Africa.
4. *Pakistani and Indian Muslim* (*n*=177): Muslim and Ismaili Muslim women, who were born/lived in Pakistan (60%), India (19%) or East Africa (20%).
5. *Bangladeshi Muslim* (*n*=35): Muslim women born in Bangladesh.
6. *Other* (*n*=4): Christian (3) and Buddhist (1) women. Due to small numbers, this group was excluded.

### Statistical methods

Conditional logistic regression models (Breslow and Day, 1980) with each set containing a case and two matched controls, were used to estimate odds ratios (ORs) and 95% confidence intervals (95% CI) associated with ethnic subgroup, with the largest group, Gujarati Hindu women, as the reference category. We tested the null hypothesis that the odds of breast cancer was constant across ethnic subgroups by comparing a model with indicators for ethnic subgroup to one without, using a likelihood ratio test (LRT). We then explored whether these associations were confounded firstly by socio-demographic, reproductive and familial breast cancer risk factors, and secondly by other markers of environmental factors. The reproductive factors were categorised as follows: age at menarche (<13, 13, 14, ⩾15 years), parity (nulliparous, 1–2, 3–4, ⩾5), age at first birth (<20, 20–24, 25–29, ⩾30 years, nulliparous), duration of breast feeding (never, <6, 6–11, 12–23, 24–47, ⩾48 months) and menopausal status (pre- or postmenopausal). Markers of familial risk were family history of breast cancer (yes/no) and parental consanguinity (yes/no). Socioeconomic variables were years of education (0, 1–7, 8–13, ⩾14 years), social class of household (defined as the higher social class of the participant and, if appropriate, her spouse/partner) and age at migration to the UK. Other markers of environmental exposures (anthropometry, diet and exercise) were treated as continuous variables.

## RESULTS

The study included 240 cases and 477 controls (for three cases it was only possible to obtain one matched control). The response rate for eligible cases that were successfully traced was 79% (240 out of 303). Of the 480 eligible controls initially selected (matched to the 240 cases), 76% (365 out of 480) agreed to participate. For those who refused, replacement controls were selected.

The odds of breast cancer varied significantly between the ethnic subgroups (LRT *P*=0.008). Pakistani/Indian Muslim women had a statistically significant 1.88-fold (95% CI: 1.19, 2.98) higher odds than Gujarati Hindu women (*P*<0.01). There was also a reduced risk (by 35%, 95% CI: −28%, 66%) in Punjabi Hindus and an increased risk (by 26%, 95% CI: −26%, 112%) in Punjabi Sikhs relative to Gujarati Hindu women, but neither of these associations were statistically significant. We investigated whether these differences were explained by known and potential breast cancer risk factors by examining (i) whether known breast cancer risk factors had associations in the expected direction; (ii) whether the distribution of these risk factors in the controls varied by ethnic subgroup and (iii) whether adjustment for these factors altered the associations of breast cancer with ethnic subgroup.

### Associations of known breast cancer risk factors

The associations of sociodemographic and reproductive variables with breast cancer were as expected (Table 1

**Table 1:** Breast cancer risk in South Asian first-generation migrants in England by sociodeomographic, reproductive and other variables: univariable analysis

	**Category per unit increase**	**OR^a^**	**95% CI^b^**	***P*-value**
*Socio-demographic characteristics*				
Age on arrival in UK	Per 5 years	1.00	0.98, 1.02	0.77
Migrated via East Africa	Yes *vs* no	1.07	0.76, 1.51	0.70
Years of education	0	1	—	—
	1–7	1.44	0.80, 2.59	0.23
	8–13	2.18	1.24, 3.83	0.01
	⩾14	2.10	1.14, 3.87	0.02
Social class	Nonmanual *vs* manual	1.64	1.16, 2.33	0.01

*Reproductive factors*				
Age at menarche (years)	Per year	0.92	0.83, 1.02	0.10
Age at first birth (years)	<20	1	—	—
	20–24	0.97	0.61, 1.55	0.91
	25–29	1.68	1.02, 2.77	0.04
	30+	1.49	0.77, 2.90	0.24
	Nulliparous	1.38	0.64, 2.96	0.42
Parity	1–2	1	—	—
	3–4	1.09	0.75, 1.59	0.64
	5–7	0.72	0.43, 1.22	0.22
	⩾8	0.44	0.12, 1.62	0.22
	Nulliparous	1.11	0.55, 2.24	0.77
Breast feeding^c^	Ever *vs* never	0.70	0.47, 1.06	0.09
Duration of breast feeding	<6 months	1	—	—
	6–11 months	1.27	0.69, 2.34	0.45
	12–23 months	0.90	0.50, 1.62	0.74
	24–47 months	1.14	0.63, 2.07	0.66
	⩾48 months	0.91	0.48, 1.74	0.77
	Never	1.50	0.92, 2.46	0.11
Menopausal status	Post *vs* pre	1.45	0.92, 2.30	0.11
Median age at menopause^d^	Per year	1.03	0.99, 1.08	0.14

*Familial factors*				
Family history of breast cancer	Yes *vs* no	1.92	0.94, 3.91	0.07
Parental consanguinity	Yes *vs* no	1.31	0.73, 2.35	0.37

*Environmental factors*				
Adult height	Per 5 cm	1.05	0.91, 1.22	0.49
Leg length	Per 5 cm	0.94	0.80, 1.10	0.43
Waist : hip ratio	Per 10%	1.05	0.88, 1.26	0.57
Waist circumference	Per 10 cm	1.19	1.03, 1.39	0.02
BMI^e^ at interview	Per 3 kg m^−2^	1.08	0.97, 1.19	0.15
MET^f^ score at age 12 years	Per 20%	0.99	0.95, 1.04	0.78
MET^f^ score at age 30 years	Per 20%	0.97	0.94, 1.01	0.20
Total energy	Per 500 kcal	1.04	0.89, 1.23	0.61
NSP^g^ per 1000 kcal	Per 2 g/1000 kcal	0.84	0.73, 0.97	0.02
% energy from fat	Per 5%	0.92	0.80, 1.05	0.22

a OR=odds ratio (estimated from a conditional logistic regression model hence controlled for age and GP).

b CI=confidence interval.

c Among parous women.

d Among postmenopausal women.

e BMI=body mass index.

f MET=metabolic equivalent score per week.

g NSP=nonstarch polysaccharides.

). Higher socioeconomic status measured either by years of education or nonmanual occupation was positively associated with breast cancer, but there was no association with age at migration to the UK. Later menarche was associated with a lower odds of breast cancer, as was younger age at first pregnancy and higher parity. Having ever breast-fed was associated with a 30% reduction in risk, which was not confounded by age at first birth or parity, though no association was observed with the total lifetime duration of breastfeeding. Risk was increased in women who had a first-degree relative with this disease, but there was no association with parental consanguinity.

A large waist circumference was statistically significantly associated with risk but MET scores at ages 12 and 30 years, total energy intake, percentage energy from fat or any other macronutrients, except nonstarch polysaccharides (NSP), were not. Women with diets high in NSP were at a statistically significant reduced risk (OR 0.54 (95% CI: 0.34, 0.88) comparing the highest to the lowest fourth of energy-adjusted NSP intake).

### Distribution of breast cancer risk factors in controls, by the ethnic subgroup

There were large variations in the distributions of potential risk factors by the ethnic subgroup among the controls (Table 2

**Table 2:** Distribution of breast cancer risk factors in controls by ethnic subgroup

	**Gujarati Hindu (*n*=186)**	**Punjabi Hindu (*n*=54)**	**Punjabi Sikh (*n*=105)**	**Pakistani/Indian Muslim (*n*=101)**	**Bangladeshi Muslim (*n*=29)**
	**Mean (SD) unless otherwise stated**				
*Socio-demographic characteristics*					
Age at diagnosis of index case (years)	51.7 (9.5)	51.9 (7.9)	50.1 (9.1)	51.8 (8.6)	52.1 (9.1)
Age at migration to UK (years)	29.2 (10.8)	25.4 (9.3)	24.8 (9.7)	28.6 (10.7)	36.6 (13.3)
% East African South Asian	70.4	14.8	21.0	24.8	0
% with no formal eduction	7.0	13.0	21.9	32.7	17.2
% in manual social classes	36.6	35.2	60	41.6	75.9

*Reproductive factors*					
Age at menarche (years)^a^	13.8 (1.8)	13.7 (1.7)	14.1 (1.7)	13.9 (1.7)	12.7 (1.4)
% nulliparous	10.2	1.4	3.7	5.7	2.9
Age at first birth (years)^b^	23.7 (4.6)	23.6 (3.8)	23.6 (4.6)	23.2 (4.4)	22.4 (4.8)
*Parity*^b^ *n* (%)					
1–2	84 (50)	24 (45)	35 (35)	25 (26)	6 (21)
3–4	69 (41)	18 (34)	45 (45)	33 (24)	5 (18)
⩾5	16 (9)	11 (21)	20 (20)	38 (40)	17 (61)
*Breast feeding*^b^					
Ever breastfed (%)	84	81	72	81	86
Median total lifetime duration (months)	12.0	5.0	7.3	12.4	63.1
Median duration per child (months)	6.5	3.7	4.4	5.1	14.5
% post menopausal	57	50	42	53	52
Median age at menopause (years)	47	47	49	46	48

*Familial factors*					
% with family history of breast cancer	4.8	3.7	3.8	3.0	0.0
% with first degree consanguineous parents	0	0	0	29.7	27.6

*Environmental factors*^c^					
Adult height (cm)	153.2 (5.6)	155.3 (6.6)	155.8 (6.3)	155.2 (6.0)	151.2 (5.4)
Leg length (cm)	75.7 (7.0)	76.0 (5.6)	75.9 (7.6)	76.6 (4.6)	73.9 (3.3)
Waist : hip ratio	0.80 (0.07)	0.81 (0.07)	0.82 (0.07)	0.83 (0.07)	0.90 (0.06)
Waist circumference (cm)	84.5 (10.0)	87.7 (10.6)	87.2 (10.2)	88.2 (11.9)	89.8 (10.9)
BMI^d^ (kg m^−2^)	27.5 (4.7)	28.7 (5.0)	28.3 (5.0)	27.9 (5.1)	26.4 (5.2)
MET^e^ score at age 12^f^	36.7 (33.5–40.3)	32.9 (27.2–39.8)	38.4 (35.0–42.2)	37.8 (32.8–43.6)	43.7 (35.5–53.7)
MET^e^ score at age 30^f^	13.4 (12.0–14.8)	12.4 (10.0–15.4)	16.0 (14.2–18.0)	16.2 (13.8–18.9)	20.8 (15.7–27.5)
% Vegetarian diet	78	43	42	0	0
Energy intake (kcal day^−1^)	1949 (488)	1923 (513)	1940 (471)	1851 (539)	1815 (469)
% Energy from fat	42.3 (6.0)	36.2 (5.5)	36.9 (4.9)	36.6 (4.9)	38.2 (5.1)
NSP^g^ (g/1000 kcal)	11.3 (1.8)	12.1 (2.3)	11.9 (2.2)	10.7 (2.4)	5.9 (2.2)

a 22 women with missing values excluded.

b Parous women only.

c All anthropometric data measured at interview; 14 women without anthropometric data excluded.

d BMI=body mass index.

e MET=metabolic equivalent score per week.

f Geometric mean and 95% confidence interval.

g NSP=nonstarch polysaccarides.

). The majority (80%) of controls migrated to the UK at or after age 20 years, but, on average, Punjabi Sikhs and Punjabi Hindus migrated at younger ages than others, particularly Bangladeshi Muslims. A large proportion (70%) of the Gujarati Hindu women migrated from or via East Africa, compared to under 25% of women in other ethnic subgroups. Gujarati and Punjabi Hindu controls had, on average, better socioeconomic circumstances compared to the other ethnic subgroups. Menarche occurred, on average, latest for Punjabi Sikh controls. Gujarati Hindu controls had the greatest percentage of nulliparity (10.2%), followed by Pakistani/Indian Muslim controls (5.7%). Among parous controls, the mean age at first birth was earlier in Bangladeshi (22.4 years) and Pakistani/Indian Muslim women (23.2 years) than in the other groups (23.6 years). The Muslim controls also had, on average, higher parity. Breast feeding was most common and of longest duration among parous Bangladeshi Muslim controls (86% had breastfed), and least common and of shortest duration in Punjabi Sikh controls (72%). The percentage of controls with a family history of breast cancer was similar in all ethnic subgroups (3–5%), with the exception of the Bangladeshi women (0%). Approximately 30% of controls in the two Muslim subgroups, but none in the Hindu and Sikh groups, had consanguineous parents.

The mean height and leg length were similar in Punjabi Hindu, Punjabi Sikh and Pakistani Muslim controls, but considerably lower in Gujarati Hindu controls and particularly so in Bangladeshi Muslim controls. Gujarati Hindu controls also had, on average, smaller waist circumferences, lower waist : hip ratio and lower BMI. MET scores at ages 12 and 30 years were lowest in Punjabi Hindu women, followed by Gujarati Hindu women, Pakistani/Indian Muslims, Punjabi Sikhs, and highest in Bangladeshi Muslims. Hindu and Sikh controls had a higher total caloric intake compared to the two Muslim ethnic subgroups. Gujarati Hindu controls, who were predominantly vegetarian, had the highest percentage energy from fat, followed by the Bangladeshi controls and then, with similar percentages, the other three subgroups. NSP intake was highest in Hindus and Sikhs, slightly lower in Pakistani/Indian Muslims and considerably lower in Bangladeshi Muslims.

### Breast cancer and ethnic subgroup

Pakistani/Indian Muslim women had a statistically significant almost two-fold increase in the odds of breast cancer compared to Gujarati Hindu women, despite their more protective reproductive profiles. Greater parity, fewer years in education and an earlier menopause, characteristic of Pakistani/Indian Muslim women (Table 2), are associated with lower risk (Table 1), hence adjusting separately for each of these resulted in higher odds ratios (Table 3

**Table 3:** Risk of breast cancer associated with ethnic subgroup, before and after adjustment for reproductive and socio-economic factors

**Ethnic subgroup (cases/controls)**	**Gujarati Hindu (80/186)**	**Punjabi Hindu (18/54)**		**Punjabi Sikh (58/105)**		**Pakistani/Indian Muslim (76/101)**		
**Controlled for age and GP (through matching) and:**	**OR^a^**	**OR^a^**	**95% CI^b^**	**OR^a^**	**95% CI^b^**	**OR^a^**	**95% CI^b^**	***P*-value^c^**
—	1	0.65	0.34, 1.28	1.26	0.74, 2.12	1.88	1.19, 2.98	0.008
Age at menarche	1	0.61	0.21, 1.19	1.29	0.76, 2.18	1.91	1.20, 3.04	0.005
Age at first birth	1	0.66	0.33, 1.30	1.28	0.75, 2.17	1.79	1.12, 2.86	0.017
Parity	1	0.68	0.35, 1.33	1.28	0.75, 2.16	2.04	1.28, 3.26	0.005
Time since menopause	1	0.70	0.36, 1.38	1.34	0.79, 2.28	1.96	1.23, 3.12	0.008
Family history of breast cancer	1	0.65	0.34, 1.28	1.24	0.73, 2.09	1.90	1.20, 3.01	0.008
Breast fed^d^	1	0.67	0.34, 1.31	1.21	0.71, 2.05	1.91	1.20, 3.04	0.009
Years of formal education^e^	1	0.64	0.32, 1.26	1.40	0.82, 2.38	2.21	1.37, 3.56	0.001
Years since migration to UK	1	0.65	0.33, 1.27	1.24	0.76, 2.10	1.89	1.19, 2.99	0.008
All of the above	1	0.74	0.36, 1.52	1.49	0.85, 2.62	2.32	1.38, 3.88	0.003

a OR=odds ratio, estimated from a conditional logistic regression model.

b CI=confidence interval.

c *P*-value for heterogeneity in odds of breast cancer by ethnic subgroup.

d Breast feeding: 0–4.4, 4.5–11, 12–23, 24–47, ⩾48 months, never.

e Years of formal education: 0, 1–7, 8–13, ⩾14 years.

). Other reproductive and sociodemographic factors did not confound this association, either positively or negatively. Thus, controlling for all the sociodemographic and reproductive factors in Table 3 resulted in an increase in the odds of breast cancer in Pakistani/Indian Muslim women compared to Gujarati Hindus from 1.88 to 2.32 (Table 3).

Table 4

**Table 4:** Risk of breast cancer associated with ethnic subgroup, before and after adjustment for various environmental factors

**Ethnic subgroup**	**Gujarati Hindu**	**Punjabi Hindu**		**Punjabi Sikh**		**Pakistani/Indian Muslim**		
**Controlled for age and GP (through matching) and:**	**OR^a^**	**OR^a^**	**95% CI^b^**	**OR^a^**	**95% CI^b^**	**OR^a^**	**95% CI^b^**	***P*-value^c^**
—d	1	0.59	0.30, 1.18	1.23	0.72, 2.11	1.76	1.10, 2.81	0.012
*Anthropometric factors*								
Adult height (cm)	1	0.59	0.30, 1.17	1.22	0.71, 2.10	1.74	1.08, 2.80	0.014
Leg length	1	0.60	0.30, 1.20	1.25	0.73, 2.14	1.78	1.11, 2.87	0.012
Waist : hip ratio	1	0.60	0.30, 1.18	1.23	0.72, 2.10	1.73	1.07, 2.79	0.017
Waist circumference (cm)	1	0.55	0.28, 1.11	1.14	0.66, 1.97	1.60	0.99, 2.59	0.024
BMI^e^ (kg/m^2^)	1	0.57	0.28, 1.14	1.21	0.70, 2.08	1.77	1.10, 2.86	0.009
								
*Physical exercise*								
MET^f^ score at age 12 (log)	1	0.60	0.30, 1.19	1.24	0.72, 2.12	1.73	1.08, 2.78	0.014
MET^f^ score at age 30 (log)	1	0.57	0.28, 1.17	1.24	0.72, 2.13	1.71	1.06, 2.78	0.017
								
*Dietary factors*								
Total energy intake	1	0.59	0.30, 1.18	1.24	0.72, 2.12	1.79	1.11, 2.87	0.011
Energy adj fat (residuals)	1	0.51	0.25, 1.05	1.11	0.64, 1.94	1.64	1.01, 2.65	0.011
Energy adj NSP^g^ (residuals)	1	0.70	0.35, 1.39	1.35	0.79, 2.32	1.51	0.93, 2.45	0.092
								
All factors in Table 3	1	0.67	0.32, 1.42	1.47	0.82, 2.61	2.12	1.25, 3.58	0.007
Table 3 factors, NSP^g^	1	0.78	0.37, 1.64	1.57	0.88, 2.82	1.82	1.06, 3.12	0.048
Table 3 factors, NSP^g^, fat	1	0.66	0.30, 1.44	1.39	0.76, 2.54	1.63	0.94, 2.85	0.079
Table 3 factors, NSP^g^, fat, waist circumference	1	0.62	0.28, 1.36	1.32	0.72, 2.42	1.49	0.85, 2.63	0.118

a OR=odds ratio, estimated from a conditional logistic regression model.

b CI=confidence interval.

c *P*-value for heterogeneity in odds of breast cancer by ethnic subgroup.

d These ORs are slightly different from those in Table 3 because the analysis in Table 4 is restricted to 97% of women who had complete dietary and anthropometric data.

e BMI=body mass index.

f MET=metabolic equivalent score per week.

g NSP=nonstarch polysaccharides.

shows the effect of adjustment for other environmental factors in the 97% of women who had complete dietary and anthropometric data. The odds ratio comparing Pakistani/Indian Muslim to Gujarati Hindu women controlled for reproductive factors (OR 2.12, 95% CI: 1.25, 3.58) was attenuated and became nonsignificant (OR 1.49, 95% CI: 0.85, 2.63) after further adjustment for waist circumference, NSP and fat intake. Thus, a greater mean waist circumference and especially a lower NSP intake, both of which are associated with an increased risk (Table 1), accounted for most of the increased odds in Pakistani/Indian Muslims.

Punjabi Sikh women showed a nonsignificant increased risk compared to Gujarati Hindu women (OR 1.26, 95% CI: 0.74, 2.12). This association was not confounded by reproductive risk factors, but was amplified after controlling for years of education and menopausal status. Controlling for all the risk factors in Table 3, the odds ratio increased from 1.26 to 1.49 (95% CI: 0.85, 2.62), but remained nonsignificant. The magnitude of this association increased further after taking the protective effect of a high mean NSP intake into account, but this was counterbalanced by the adverse effect of a larger waist circumference. Thus, the odds ratio adjusted for reproductive factors, waist circumference, NSP and fat intake was 1.32 (95% CI: 0.72, 2.42).

By contrast, Punjabi Hindu women had a nonsignificant 35% reduction in the odds of breast cancer compared to Gujarati Hindu women, although the confidence interval is very wide due to only 18 cases. The magnitude of this reduction decreased slightly with adjustment for the reproductive and socioeconomic factors in Table 3, but resumed its original level after further adjustment for NSP and fat intake and waist circumference (Table 4).

The univariable analysis showed that Bangladeshi women were at a much reduced odds compared to all other ethnic subgroups (OR 0.33 (95% CI: 0.10, 1.06) compared to Gujarati Hindu women). This reduction is possibly driven by their lower mean age at first birth, higher parity and longer lifetime duration of breast-feeding (Table 2), but as this subgroup consisted of only six cases and 29 controls, no further analyses were possible.

## DISCUSSION

We endeavoured to obtain a representative sample of the South Asian population in England by using population-based controls, the ethnic distribution of which was similar to that expected according to the 1991 Census. The observed/expected percentage of controls by ethnic subgroup was 77%/78%, 18%/14% and 6%/9% for Indians, Pakistanis and Bangladeshis, respectively (dos Santos Silva *et al*, 2002). Risk factor distributions in controls were also similar to that in Census and Health Survey data. For example, that a higher percentage of Pakistanis (45%) compared to Indians (31%) had no qualifications in the 2001 census (Office for National Statistics, 2003) is consistent with our findings for educational attainment. Face-to-face interviews with each participant should have resulted in minimal misclassification of ethnic subgroup. By matching on GP, we may have made controls similar to cases with respect to ethnic subgroup, as first-generation migrants from the same region of origin often settle in particular areas, but this would have led to an attenuation of the results and so would not explain our findings.

We found great heterogeneity in breast cancer risk between South Asian ethnic subgroups, with risk increasing in the following order: Bangladeshi Muslims (OR=0.33), Punjabi Hindu (0.65), Gujarati Hindu (1=reference group), Punjabi Sikh (1.26) and Pakistani/Indian Muslims (1.88).

Pakistani/Indian Muslims were the only group with a statistically significant increased risk compared to Gujarati Hindu women. Their increased risk is consistent with a study of all deaths in 1975–77 in England and Wales, in which proportional mortality ratios were much higher in Muslims compared to Gujarati and Punjabi South Asian female immigrants (Balarajan *et al*, 1984). Additionally, similar associations were found in Greater Bombay in 1973–78 where breast cancer incidence rates were highest among Parsi women, followed by Christian, Muslim and then Hindu women (Jussawalla *et al*, 1985). Breast cancer incidence rates reported by cancer registries in the Indian subcontinent also suggest higher rates in Pakistani women, being 51.7 per 100 000 women in 1995–97 in Karachi, Pakistani (Bhurgri *et al*, 2000) compared to 24.9 and 25.1 in the Indian cities of Bombay in 1985 (Yeole *et al*, 1990) and Calcutta in 1998–99 (Sen *et al*, 2002), respectively. This study therefore suggests that the ranking of risk among South Asians in the UK remains the same as that in the subcontinent, despite higher overall incidence rates in the UK (Winter *et al*, 1999).

The major benefit of this study is the opportunity to investigate factors that might account for the differences in risk. Reproductive factors have previously been shown to have similar associations in low- and high-risk areas (Rao *et al*, 1994), as was the case in the present study. However, although having breastfed was protective, the lack of association with the duration of breast feeding is contrary to the finding of a large pooled analysis (Collaborative Group on Hormonal Factors in Breast Cancer, 2002) and may be due to nondifferential recall bias. A high intake of NSP was associated with lower breast cancer risk, as we previously reported (dos Santos Silva *et al*, 2002). The increased risk associated with a large waist circumference is consistent with findings from other studies (Huang *et al*, 1999; Friedenreich *et al*, 2002). Waist circumference was, however, measured after diagnosis for cases so may have been affected by the disease and/or its treatment (i.e. by chemotherapy (Aslani *et al*, 1999)), but when analyses were restricted to women who reported not having changed weight in the past 2–3 years, this association strengthened further. We could not explain the lack of association of adult height with breast cancer by too homogeneous a height distribution, as the interquartile range (139–169 cm) was as wide as that in studies that have found an association (van den Brandt *et al*, 1997). Rates of consanguineous marriages in parents of Pakistani/Indian Muslim controls (30%) were similar to those observed in Pakistan (Bittles *et al*, 1993), but this factor was not related to breast cancer in this study.

The almost two-fold greater risk of breast cancer in Pakistani/Indian Muslims than in Gujarati Hindus is somewhat surprising given their larger family sizes, earlier age at first birth and longer duration of breast feeding; thus, taking these factors into account widened the difference further. This association was unexplained by markers of genetic influences such as family history of breast cancer or parental consanguinity.

These findings seem to indicate that much of the difference in risk is due to lifestyle/environmental factors other than reproductive behaviour. Firstly, a lower intake of NSP in Pakistani/Indian Muslim women compared to Gujarati Hindu women accounted for much of the increased risk. Pakistani women tend to be meat-eaters, whereas most Hindus and many Sikhs are lacto-vegetarians. Gujarati Hindu controls consumed more vegetable dishes (mean 347 g day^−1^), more pulse/lentil/dhal dishes (103.2 g day^−1^) and more fruit (1.8 servings day^−1^) compared to Pakistani/Indian Muslim women (with corresponding intakes of 256, 40.7 g day^−1^ and 1.6 servings day^−1^, respectively), resulting in their higher NSP intake. These intakes are consistent with a survey in pregnant women (Wharton *et al*, 1984) and with the Health Survey for England (2001), in which, for example, 28% of Indian women consumed vegetables at least six times per week, compared to 11% of Pakistani women. Greater differences in fibre intake exist in the Indian subcontinent (FAO, 1982); therefore, these groups may have had even greater differences in NSP intake before migration and, in particular, during childhood, a period of aetiological importance for breast cancer (Hilakivi-Clarke *et al*, 2001). Secondly, a larger waist circumference (by 3.7 cm compared to Gujarati Hindu controls (standard error 1.3 cm)) also contributed to their increased risk. This possibly reflects their more sedentary lifestyles, as only 28% of Pakistani/Indian Muslim controls had worked outside the home in the 10 years prior to interview, whereas two-thirds of Hindu and Sikh women had. The Health Survey in England (2001) also found lower levels of physical activity in Pakistani women, for exmaple, 72% of Pakistanis compared to 39% of Indian women aged 55 years and over had not participated in any form of moderate/vigorous activity in the last 4 weeks. However, MET scores based on recall of activities at ages 12 and 30 years were not related to breast cancer in this study and did not differ across the ethnic subgroups.

Punjabi Sikh women, the second largest Indian ethnic subgroup in the UK, were at an increased, although nonsignificant, risk of breast cancer compared to Gujarati Hindu women, after adjusting for known risk factors. In contrast, Punjabi Hindu and Bangladeshi Muslim women had a lower (nonsignificant) risk relative to Gujarati Hindu women. The distinct characteristics of the Bangladeshi Muslim subgroup, such as a fish-based diet, short stature and favourable reproductive profile, may influence breast cancer risk and will make this an interesting group to study in future years as more of this population reaches the ages when breast cancer risk is highest.

In summary, this study reveals that breast cancer rates for South Asian women in England mask great heterogeneity in risk within this diverse ethnic group. Pakistani/Indian Muslim women were at the highest risk compared to all other South Asian ethnic subgroups – an association that appears to be related to differences in diet and body size. These findings may be of use when considering the low uptake of breast screening in South Asian women (Hoare, 1996). They suggest that characterising all South Asians, regardless of their ethnic subgroup, as having a ‘low risk’ of breast cancer relative to non-South Asians may be misleading, particularly in light of recent findings showing that breast cancer incidence is rising faster among South Asians in England than among non-South Asians (Smith *et al*, 2003). This study highlights the need for further research on breast cancer within the South Asian population and, in particular, the need for estimation of absolute incidence risks by ethnic subgroup.
